# Cytoskeletal Disruption after Electroporation and Its Significance to Pulsed Electric Field Therapies

**DOI:** 10.3390/cancers12051132

**Published:** 2020-04-30

**Authors:** Philip M. Graybill, Rafael V. Davalos

**Affiliations:** 1 BEMS Lab, Department of Biomedical Engineering and Mechanics, Virginia Tech, Blacksburg, VA 24061, USA; davalos@vt.edu; 2 Department of Mechanical Engineering, Virginia Tech, Blacksburg, VA 24061, USA; 3 Virginia Tech–Wake Forest University, School of Biomedical Engineering and Sciences, Blacksburg, VA 24061, USA

**Keywords:** pulsed electric fields, electroporation, cytoskeleton, actin, microtubules, intermediate filaments, cell junctions, nsPEFs, IRE, ECT, mechanobiology, vascular lock, cancer

## Abstract

Pulsed electric fields (PEFs) have become clinically important through the success of Irreversible Electroporation (IRE), Electrochemotherapy (ECT), and nanosecond PEFs (nsPEFs) for the treatment of tumors. PEFs increase the permeability of cell membranes, a phenomenon known as electroporation. In addition to well-known membrane effects, PEFs can cause profound cytoskeletal disruption. In this review, we summarize the current understanding of cytoskeletal disruption after PEFs. Compiling available studies, we describe PEF-induced cytoskeletal disruption and possible mechanisms of disruption. Additionally, we consider how cytoskeletal alterations contribute to cell–cell and cell–substrate disruption. We conclude with a discussion of cytoskeletal disruption-induced anti-vascular effects of PEFs and consider how a better understanding of cytoskeletal disruption after PEFs may lead to more effective therapies.

## 1. Introduction

In recent years, pulsed electric fields (PEFs) have become an important clinical tool for the treatment of tumors by Irreversible Electroporation (IRE) [1,2,3,4,5] and Electrochemotherapy (ECT) [6,7]. Clinical and preclinical studies of nanosecond PEFs (nsPEFs) [8,9], Gene Electotransfection (GET) [10,11,12], and electrofusion [13,14] therapies show significant potential for cancer treatment. Beyond cancer therapy, PEFs are useful for a variety of purposes including bacterial inactivation [15,16], decellularization of tissues [17,18], extraction of biomolecules [19,20], and numerous GET applications [21,22,23,24,25,26]. Exogenous electric fields applied as short, high-magnitude pulses cause electroporation, a phenomenon characterized by increased cell membrane permeability. Classical electroporation theory describes metastable, lipidic pores formed by PEFs that enable uncontrolled molecular and ionic transport across the cell membrane and cause a loss of cell homeostasis [27]. Additionally, modulation of voltage-gated ion channels and oxidization of lipids can further increase membrane permeability after PEFs [28]. PEF therapies such as IRE and nsPEFs rely on PEF-induced cell disruption to destroy tumor cells. ECT combines reversible PEF disruption with adjuvant chemotherapy to enhanced drug uptake and cause cell death. Likewise, GET combines reversible PEF disruption with nucleic acids to enhance the transfection of cells for therapeutic purposes. Several excellent reviews are available on electroporation theory [27,28] and PEF therapies in clinical/preclinical oncology [6,29,30,31,32].

**Figure 1:**
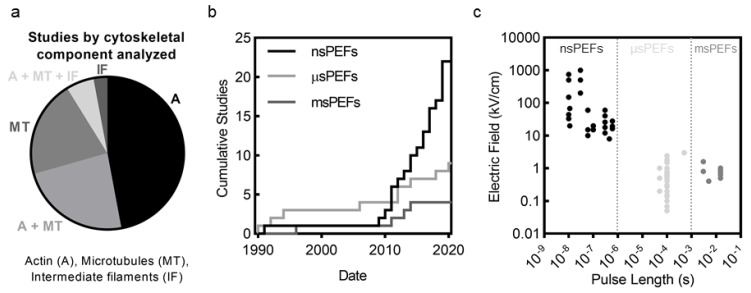
An analysis of published studies since 1990 on cytoskeletal disruption by pulsed electric fields (PEFs). (**a**) Actin disruption is the cytoskeletal component most frequently investigated by studies. Many studies also consider microtubules (MT) disruption. Few studies, however, consider disruption to intermediate filaments (IFs) and no studies consider septin disruption. (**b**) Since 2010, there has been significant interest in nanosecond PEF (nsPEFs), which now account for over half of all studies on PEF-induced cytoskeletal disruption. Microsecond PEFs (µsPEFs) and millisecond PEFs (msPEFs) have also seen an increase in studies. (**c**) Studies cover a wide range of pulse lengths and field magnitudes. nsPEFs are applied at high field strengths (generally >10 kV/cm), while µsPEFs and msPEFs are applied at lower (0.1–2 kV/cm) field strengths. Data points show field strengths tested in these studies.

In recent years there has been a growing appreciation that the cell cytoskeleton is involved with and affected by PEFs [33]. The cell cytoskeleton, composed of actin, microtubules (MT), intermediate filaments (IFs), and septin, provides structure and mechanical stability to cells, enabling tensional homeostasis with the cell’s environment [34,35]. Critical cell functions such as proliferation, differentiation, signaling, migration, and cell survival would not be possible without the cell cytoskeleton [36,37]. These filamentous structures dynamically adapt to control intracellular transport, organelle location, cell contractility, cell shape, cell volume, and cell behavior, among many other functions. Cytoskeletal filaments provide support to the highly fluid, flexible, and extensible plasma membrane through linker proteins, that together enable mechanical interactions with adjacent cells via cell–cell junctions or with the environment via cell–substrate adhesions. Of the studies surveyed in this review, the majority focus on actin and MTs, with few studies considering disruption to IFs and no studies considering disruption to septin (Figure 1a).

The number of studies investigating cytoskeletal disruption has increased dramatically in the last decade (Figure 1b). In particular, nanosecond PEFs (nsPEFs) have seen tremendous growth in the number of studies and now account for over half the studies on PEF-induced cytoskeletal disruption. Studies on cytoskeletal disruption include a broad range of pulse lengths, from nanosecond pulses (nsPEFs) to millisecond PEFs (msPEFs) (Figure 1c). Microsecond and millisecond PEFs such as those used for IRE, ECT, and GET are generally applied at electric field strengths between 0.1 and 2 kV/cm. In contrast, nsPEFs generally employ field strengths over 10 kV/cm. (Figure 1c). These high field strengths and short pulse lengths of nsPEFs cause smaller pore radii than longer pulses [9,38,39], phosphatidylserine externalization [40,41], elevated intracellular calcium concentration [42,43,44], depolarization of mitochondria [45,46,47], DNA damage [42,48,49], altered ion channel activity [50], and apoptosis [9,51]. Results from studies investigating cytoskeletal disruption seem to indicate that most observed features of cytoskeletal disruption are conserved across pulse lengths ranging from nsPEFs to msPEFs. Pulse length and magnitude, however, likely do influence the mechanisms of disruption (see Section 5.7) and are important factors in understanding cell response.

In this review, we seek to provide a comprehensive summary of studies investigating cytoskeletal dynamics after PEF treatment. To this end, we have included a table that summarizes the study parameters and key findings of relevant studies, arranged by pulse length (Table 1). Beginning our discussion with actin, we briefly review the cytoskeletal structure before summarizing descriptions of cytoskeletal changes after PEFs. We then review suggested mechanisms of cytoskeletal disruption, and discuss how cytoskeletal disruption impacts cell–cell and cell–substrate interactions. Finally, we discuss the significance of cytoskeletal disruption to electroporation therapies, focusing on anti-vascular effects, and end our review with an outlook on how understanding cytoskeletal disruption may improve PEF therapies.

## 2. Actin and PEF-induced Actin Disruption

Evolutionarily conserved and highly abundant in cells, actin is a key component of the cell cytoskeleton. Actin monomers polymerize spontaneously to form thin (~7 nm-diameter), flexible, helical filaments composed of two strands of actin subunits [52]. Actin polymerization preferentially occurs at the plus end of an actin filament, and filament dynamics are controlled by a variety of regulatory proteins that nucleate new filaments, elongate existing filaments, terminate filaments, sever filaments, crosslink filaments, and sequester unpolymerized actin monomers. Hydrolysis of actin-bound ATP occurs randomly along filaments and leads to depolymerization.

**Figure 2:**
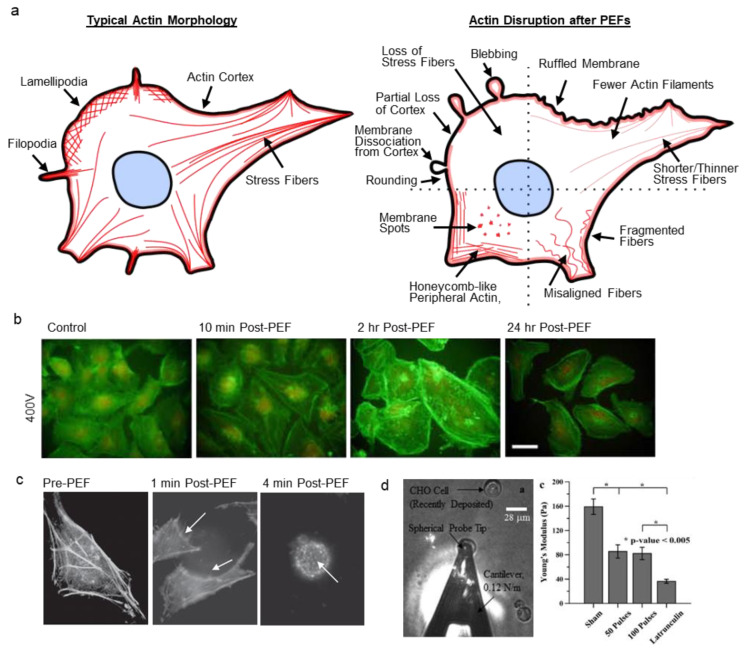
PEF-induced actin disruption. (**a**) Normal actin structures include stress fibers, filopodia, lamellipodia, and cortical actin (left). PEF-induced cytoskeletal disruption takes on many forms, such as a loss of f-actin, cell rounding, membrane ruffling, actin spots/foci/podosomes, dissociation of the cortex from the membrane, and blebbing. (right) (**b**) Actin disruption of a human dermal microvascular endothelial cell (HMEC-1) monolayer after eight, 100 µs PEFs. Scale bar 50 µm. Adapted from [53]. (**c**) Human cervical cancer (HeLa) cells after a single 60 ns PEF showed membrane ruffling (center), cell rounding (right), and actin spots on the membrane (right). Adapted with permission from [49]. (**d**) Atomic force microscopy (AFM) measurements show cell elasticity decreases after PEFs. The Young’s modulus (YM) of Chinese hamster ovary (CHO-K1) cells decreased ~50% after 10 ns PEFs (50 and 100 pulses). Adapted with permission from [54].

Actin fibers generally interact to form networks or bundles that regulate cell shape, locomotion, intracellular transport, enable cell–cell and cell–matrix interactions, and define the cell’s mechanical properties. In a cell, normal actin features include stress fibers, lamellipodia, filopodia, microvilli, and retraction fibers (Figure 2a) [36]. Importantly, actin forms the cell cortex, a dense network of crosslinked filaments beneath the lipid bilayer. Cortical actin connects to the plasma membrane via linker proteins to provide mechanical support to the otherwise highly extensible and compliant lipid bilayer. To create contractile forces, aligned and bundled actin filaments form stress fibers and use ATP-driven myosin motors to contract. Compared to muscle cells, actin contractility is more dynamic in non-muscle cells and enables cells to quickly adapt their structure to changing conditions. Additionally, actin links to cell–cell junctions such as adherens and tight junctions, as well as to cell–substrate adhesion complexes such as focal adhesions. The numerous, dynamic roles of actin allow PEF-induced disruption to take on many forms.

### 2.1. Actin Disruption

Visual manifestations of actin disruption after PEFs (Figure 2a–c) vary significantly between studies. For example, actin filaments have been reported to become shorter [55], thinner [53,56], fragmented [53,57], less densely spaced [56,57], misaligned [55,57], depolymerized [57,58,59], and show decreased fluorescence [49,55] after PEFs. Some studies report actin localization to the cell periphery [56] and honeycomb-like f-actin patterns [53,60] after PEFs. Actin contraction and detachment of trans-vacuolar actin bundles in plant cells were also observed [58]. Several studies report diminished f-actin features concomitant with an increase in background fluorescence, presumably indicating more oligomeric actin [55]. After electroporation, cells have been reported to have ruffled membranes [49,53,59], microvilli [61,62,63], form podosomes [56], show bright actin spots (foci) on the surface of cells [49,64], or show a partial loss of the actin cortex [54,64]. Some studies report a loss of membrane bright spots and homogenous actin [65]. Cell rounding and shape change are also frequently reported [49,55,56,59,64,65,66]. Unique features, such as pseudopod-like blebs, have also been demonstrated after PEFs [67]. Yet, at least one study reported no changes to actin after PEFs [68].

Factors that cause these cytoskeletal alterations are discussed in Section 5, but the diversity of disruption characteristics reported by studies is at least in part due to cell type, pulsing media, and pulse type (duration, magnitude). For a complete list of studies evaluating actin disruption after PEFs, see Table 1. Table 1 includes information such as cell type, cell attachment (adherent, suspension, monolayer), pulsing parameters, and key cytoskeletal findings from each study, and is arranged by pulse length. We briefly highlight a few studies indicating actin disruption in the following paragraphs.

In a study by Stacey et al., actin-stained human cervical cancer (HeLa) cells showed membrane ruffling and reduced staining intensity within one minute after a single 60 ns pulse (60 kV/cm) (Figure 2c) [49]. By 3–4 min, cells rounded and showed membrane speckling (bright spots). Human T lymphocytes (Jurkat cells) were more susceptible to PEF treatment than HeLa cells, and showed speckled membrane morphology within seconds but did not recover.

Chopinet et al. showed that PEF-treated (5 ms, 0.4 kV/cm, 8 pulses (p)) Chinese hamster ovary (CHO wild type) cells had membrane rippling, fewer membrane extensions, and a smoother, more homogeneous membrane 3–15 min after PEFs [59]. Stress fibers were disrupted and were not visible between 15–23 min. By 23 min after pulsing, cells re-spread and no longer showed membrane rippling. Membrane permeability was reduced to baseline levels within 10 min of pulsing, demonstrating that cytoskeletal disruption continued after membrane resealing. Cell swelling was observed. This study used atomic force microscopy (AFM) and found that cell stiffness decreased after PEFs, a topic that is discussed in Section 2.1.1.

Application of 100 ns pulses (20 kV/cm, 20 p) by Steuer et al. to rat liver epithelial (WB-F344) cells caused actin reorganization within five minutes [55]. In contrast to well-aligned actin stress fibers spanning the cell before PEF treatment, five minutes after PEFs actin fibers appeared shorter and less aligned. After 15 min, few well-defined fibers were visible, but instead, an increase in diffuse background intensity throughout the cell was seen, indicating oligomeric actin in the cytosol. Actin filaments began to reform by 30 min, and partial recovery was achieved by 60 min. The study found that the recovery timescale of the actin cytoskeleton did not correlate with temporal changes in Young’s modulus (YM).

Meulenberg et al. studied actin disruption in a human dermal microvascular endothelial cell (HMEC-1) monolayer after PEFs and ECT treatment (100 µs, 0.07–0.7 kV/cm, 8 p) over a range of field strengths (Figure 2b) [53]. Cells showed f-actin fibers spanning the cells, which formed an intact monolayer before pulsing. Ten minutes after low-magnitude PEFs, f-actin remained intact but granules and spots formed on the outer membrane. At the same timepoint, high fields resulted in less-rigid and fragmented actin, cell swelling, and peripheral actin in a honeycomb-like appearance. Two hours after PEFs, cells continued to show swelling and f-actin was significantly recruited to the cell periphery. ECT decreased cell turgidity and caused a spindle-like morphology. Monolayer recovery was achieved for low-level PEFs by 24 h, but high-magnitude PEF and ECT treated cells did not recover.

In another study by Kanthou et al. with a monolayer of human umbilical vein endothelial cells (HUVECs), actin fibers dissolved immediately (<5 min), and staining showed diffuse background fluorescence after 100 µs PEFs (0.05–0.2 kV/cm, 3 p) [60]. Fine processes extended away from the cell periphery, and actin acquired a honeycomb-like appearance. Actin regained its structure within 1 to 2 h. Western blotting indicated that phosphorylated myosin light chain (pMLC), which mediates actomyosin contraction, decreased immediately after PEFs but then dramatically increased in a burst at 30 and 60 min. The total level of actin was unaffected for a least 16 h.

#### 2.1.1. Actin-Induced Cell Elasticity Changes

Actin has been shown to be the primary cytoskeletal component contributing to cell elasticity, or Young’s modulus (YM) [85,86,87]. Thus, changes to the actin cytoskeleton can be investigated by atomic force microscopy (AFM). Several studies show that YM significantly decreases after PEFs. In a study by Chopinet et al., the YM of adherent CHO (wild type) cells decreased across the cell within one minute, and by 8 min was only 60% of pre-PEF values after millisecond PEFs (5 ms, 0.4 kV/cm, 8 p) [59]. During this time, cell elasticity became homogenous across the cell. Recovery of heterogeneous elasticity (areas of high stiffness) began by 17 min, and YM slowly returned to pre-pulse values by 35 min. Nanosecond PEFs also decrease YM. Steuer et al. found that the elasticity of rat liver epithelial (WB-F344) cells decreased by approximately one third across the cell periphery and 60% above the cell nucleus at 8 min after 100 ns PEFs (20 kV/cm, 20 p) [55]. Recovery of elasticity occurred between 13–28 min, despite incomplete f-actin recovery at these timepoints. In this study, cells did not uptake propidium iodide (PI) and did not show signs of swelling. In a study by Dutta et al., a single 60 ns PEF at 15 kV/cm caused little morphological changes in human T lymphocytes (Jurkat Clone E6-1), however, YM decreased over 50% when these cells were fixed immediately after PEF treatment [64]. At 60 kV/cm, cell shape was altered, peripheral actin became more diffuse, foci appeared, and YM decreased 85%. In another study by Thompson et al., even very short 10 ns PEFs (150 kV/cm, 100 p) decreased YM 50% on relatively round, newly-adherent Chinese hamster ovary (CHO-K1) cells 90 min after PEFs and imaging revealed a partial loss of the actin cortex (Figure 2d) [54].

Due to the short indentation depths used in some of these studies, decreased YM points to dissociation of the plasma membrane from the actin cortex due to a loss of lipid anchoring [59]. These studies also show partial loss of the actin cortex and loss of f-actin fibers, which may contribute to decreases in YM. Inhibiting actin polymerization with chemical agents decreases YM similarly to PEF treatment [69].

#### 2.1.2. Actin, Membrane Permeability, and Cell Viability

There is strong evidence that cytoskeletal proteins or associated proteins affect membrane permeability, particularly pore expansion and resealing. For example, the PEF treatment of giant unilaminar vesicles (GUVs) results in micron-diameter macropores. Macropores, however, do not form on PEF-treated cells or actin-encapsulated GUVs [72]. The lack of macropores in cells suggests pore expansion is limited by the cytoskeleton and associated proteins [88]. Secondly, experiments with cells demonstrate long-lived permeability after PEFs (minutes–hours) [59,61,89,90,91], a result that contradicts molecular dynamics simulations [92,93,94,95] and lipid vesicle experiments [96,97] that show purely lipidic pores reseal rapidly (nanoseconds–milliseconds). Comparted to empty GUVs, actin-encapsulated GUVs show significantly prolonged permeability [72]. Likewise, agar-encapsulated GUVs show extended permeability compared to fluid-filled GUVs because impingement of the membrane on the agar structure stabilizes pores [98]. Electropermeabilized regions of the cell are also not laterally mobile [99], suggesting cytoskeletal involvement.

To understand how actin structure impacts cell permeability and viability post-PEFs, several studies pretreated cells with actin-disrupting agents prior to PEF treatment. Table 2 lists agents used to disrupt actin and the studies using these agents. Use of these agents requires careful attention to concentration and exposure because these agents can be toxic, and thus deleterious to cell viability and permeability on their own. Given the reported data, it is unclear whether inhibition of actin polymerization is deleterious or protective against PEFs.

Several studies show that exposure to actin-inhibiting drugs before PEFs increases membrane permeability and decreases cell survival after PEF treatment. In a study by Rols et al., the permeability of Chinese hamster ovary (CHO-WTT clone) cells exposed to cytochalasin B (2, 20, 60 µM; 30 min incubation) caused extended permeability and significant cell death after PEF treatment (100 µs, 1.8 kV/cm, 10 p) [61]. Likewise, in a study Stacy et al., two cell lines pretreated with cytochalasin B (10 µM; 30–40 min) had decreased viability after a single 60 ns PEF (60 kV/cm) compared to PEF treatment alone [49]. Thompson et al. showed a similar result in two studies using 10 ns PEFs (150kV/cm), finding that latrunculin A pretreatment (1.2 µM; 1 hour) increased PI uptake and Annexin V-FITC signal after PEFs, and decreased cell viability of CHO-K1 cells compared to PEF treatment alone [54,83]. These studies suggest disruption of the actin cortex leads to extended permeability and decreased post-PEF viability.

Other studies, however, report the protective effects of actin disruption before PEF treatment. In a recent study by Kim et al., pretreatment with cytochalasin D (0.2–2 µM; 1 hour) significantly decreased PI uptake in human lung carcinoma cells (NCI-H460) and normal human lung fibroblasts (MRC-5) after 100 µs PEFs (0.3–1 kV/cm, 8 p) [74]. Pretreatment with low levels of cytochalasin D (0.2–0.4 µM) followed by low-magnitude PEFs also decreased Annexin V-FITC signal. In another study by Xiao et al., disruption of actin in human hepatocellular carcinoma (HepG2) cells with cytochalasin B (2 µM; 30 min) prior to PEFs (450 ns, 8 kV/cm, 30 p) led to fewer apoptotic and necrotic cells (higher overall viability) than undisrupted cells treated with PEFs [78]. In this study, early apoptotic cells decreased from 17 ± 3% to 5 ± 3% as indicated by Annexin+/PI− cells, and necrotic cells decreased from 21 ± 3% to 14 ± 3%. Cytochalasin B inhibited the loss of mitochondrial membrane potential after nsPEFs, suggesting that actin disruption may prevent signal transduction from the membrane to the mitochondria and thus hinder apoptosis. Finally, Berghofer et al. showed that in a tobacco cell line (BY-2), pretreatment with phalloidin (1 µM; 30 min) before 10 ns PEFs (33 kV/cm, 1 p) suppressed actin detachment from the cell periphery and reduced trypan blue uptake [58].

Additional studies are needed to elucidate the factors that contribute to the differential responses of cells to PEF treatment after actin disruption. Cell type, drug type, and drug exposure (concentration and duration) likely have a significant influence on study outcomes. Pulse length and pulse magnitude also may be partly responsible for the differential outcomes.

Actin stabilizing drugs have shown little effect on cell response to PEFs. Thomson et al. found that chemically stabilizing actin by jasplakinolide (10 nM; 1 hour) before PEF treatment did not change the PI and Annexin V-FITC fluorescence of CHO-K1 cells compared to PEF treatment alone (10 ns, 150 kV/cm, 100 p) [83]. In other investigations by Rols et al. and Teissie et al., the addition of ATP and GTP (1 mM) to the pulsation buffer did not change membrane permeability or resealing times of CHO-WTT clone cells after 100 µs PEF treatment (1.8 kV/cm, 10 p) [61,62].

## 3. Microtubules and PEF-Induced Microtubule Disruption

**Figure 3:**
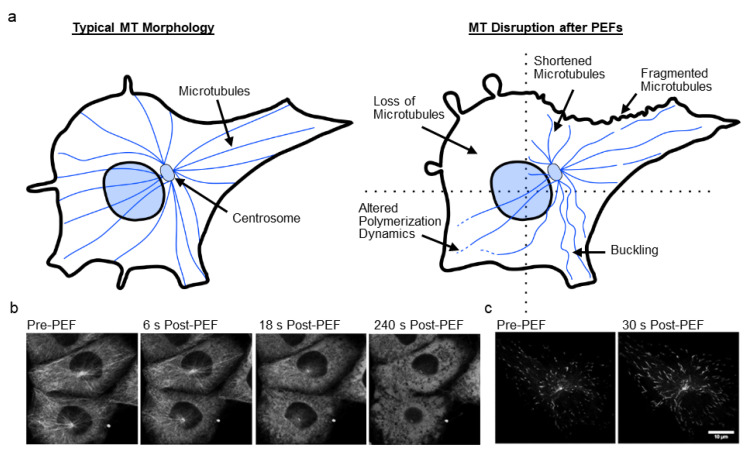
PEF-induced microtubule (MT) disruption. (**a**) MTs nucleate from the centrosome in mammals, and radiate (polymerize) outward under normal conditions (left). After PEFs, MTs may show buckling, fragmenting, altered growth, or be depolymerized (right). (**b**) Chinese hamster ovary (CHO-K1) cells show depolymerization of MTs after 600 ns PEFs in calcium-containing media. Adapted from [75]. (**c**) MT end-tracking protein EB3 demonstrates altered MT dynamics in human glioblastoma (U-87 MG) cells after 10 ns PEFs. Both the rate of polymerization and the number of polymerizing MTs change. Adapted from [47].

Microtubules (MTs) are composed of α- and β-tubulin heterodimers that stack together, forming 13 protofilaments that create a hollow, cylindrical tube-like structure of approximately 25 nm-diameter [37,100]. Unlike actin, MTs are relatively stiff [101,102]. MTs nucleate on the centrosome (in animal cells) near the nucleus and radiate outward toward the cell periphery (Figure 3a) [103]. MTs exhibit dynamic instability, rapidly switching between polymerization and depolymerization at their plus ends [104]. Growing MTs assemble GTP-bound tubulin at their tips creating a cap, that if depleted by GTP hydrolysis causes catastrophic depolymerization as protofilaments peel away from the walls [105]. Many MT-associated proteins regulate tubulin polymerization [106].

MTs play an important role in mitosis, meiosis, intracellular transport, and cell mobility [37]. Motor proteins kinesins and dyneins power intracellular transport with ATP [107], and control the positions of intracellular organelles [108].

### 3.1. Microtubules Disruption

MT disruption after PEF treatment has been less studied than actin, but MTs have been reported to fragment [47,53,60], buckle [47], become less extended [53], become densely packed [53], be depolymerized [68], and change polymerization dynamics [47] (Figure 3a). The timescale of MT recovery is similar to that of actin, with recovery in a few hours [60,68]. Table 1 lists studies investigating MT disruption along with key findings of these studies. A few of these studies are highlighted below, while others are discussed in Section 5 on the mechanisms of cell disruption.

Harkin et al. showed that chick embryo corneal fibroblasts treated with 10–20 ms PEFs (0.5–1.0 kV/cm, 1 p) in serum-free media lost their dense network of radiating MTs, with few MTs remaining after 10 min [68]. Short MTs were present after 1 hour, and complete recovery of MTs occurred within 3–4 h.

In a study by Kanthou et al., 100 µs PEFs (0.05–0.2 kV/cm, 3 p) delivered to a monolayer of human umbilical vein endothelial cells (HUVECs) caused MT disruption in the form of MT fragmentation and depolymerization [60]. At sufficient voltages, complete loss of MTs occurred. Recovery of MT structure occurred in 1–2 h post-PEFs. Total β-tubulin concentration remained constant for up to 16 h after PEFs.

In another study by Thompson et al., cells treated with 600 ns PEFs (16.2 kV/cm, 20 p) showed MT depolymerization within seconds when the pulsation buffer contained calcium (Figure 3b), even in buffer supplemented with polyethylene glycol to mitigate swelling and blebbing [75]. Pulsation buffer with calcium resulted in inhibited lysosomal transport as MT structures depolymerized, whereas lysosomes were mobile and MT disruption was less pervasive in calcium-free media.

A recent study by Carr et al. showed that 10 ns pulses (44 kV/cm, 100 p) delivered to human glioblastoma (U-87 MG) cells caused MT buckling, breakage, depolymerization, and altered MT dynamics as indicated by GFP-labelled end-tracking protein EB3 (Figure 3c) [47]. After PEFs, the number of EB3 comets decreased, and comet length increased. Tubulin and EB3-GFP accumulated near the plasma membrane and decreased in fluorescence. These observations suggest a reduced number of nucleating/growing MTs, but a faster growth rate. In this study, MT disruption was temporally associated with loss of mitochondrial membrane potential, and EB3 changes were found to be independent of calcium and cell swelling, suggesting a direct breakdown of interphase MTs. A subsequent study by Havelka et al. using 11 ns pulses (~67.5 kV/cm, 4000 p) also showed significant changes to EB3 dynamics in rat basophilic (RBL-2H3) cells [82].

### 3.2. Microtubules, Membrane Permeability, and Cell Viability

Inhibiting MTs may decrease membrane permeability to provide protective effects from PEFs, according to some studies. Table 2 lists MT-disrupting agents and studies using these agents. In two similar studies by Rols el al. and Teissie et al., half of CHO-WTT clone cells treated with 100 µs PEFs (1.8 kV/cm, 10 p) resealed within 6 min, but inhibiting MTs by pretreatment with colchicine (6.3 µM, 30 min) decreased this resealing time to within 2 min [61,62]. In another study by Rols et al., CHO-WTT clone cells that were pretreated with colchicine (6.3 µM, 30 min) before 100 µs PEFs (1.5 kV/cm, 1 p) showed decreased electrofusion events, suggesting MT involvement with membrane dynamics [63]. In a study with 10 ns PEFs (150 kV/cm, 100 p) by Thompson et al., CHO-K1 cells treated with nocodazole (10 µM; 1 hour) had less PI uptake and reduced Annexin V-FITC fluorescence, suggesting that chemically disrupting MTs might interfere with cell signaling and prevent additional membrane damage [83].

As with actin stabilization, MT-stabilizing drugs have shown little effect on cell response to PEFs. Thompson et al. found that stabilization of MTs by paclitaxel (7.5 nM; 1 hour) did not change the amount of damage to the cell membrane after 10 ns PEFs (150 kV/cm, 100 p) in CHO-K1 cells [83]. Additional studies, however, are needed to fully characterize the effects of MT agents on cell response to PEFs.

## 4. Intermediate Filaments and Septins

Intermediate filaments (IFs) assemble from fibrous subunits in a coil-coiled configuration to create rope-like filaments of high tensile strength. These filaments are dynamic and flexible, with an average diameter of about 10 nm [109]. IFs resist mechanical stresses by crosslinking other filaments within the cell and anchoring to desmosomes on the plasma membrane. IFs are composed of a family of related proteins having a common structure and can be broadly grouped into four categories: keratin filaments, vimentin and vimentin-related filaments, neurofilaments, and nuclear lamins [110].

Disruption of IFs after PEFs has not been well-studied, however, studies show IFs can be disrupted by PEFs. Harkin et al. found a perinuclear collapse of vimentin intermediate filaments following 10–20 ms PEFs (0.5–1.0 kV/cm, 1 p) in chick embryo corneal fibroblasts [68]. Recovery of vimentin fibers paralleled MT recovery, with full recovery in about 3–4 h. In a study by Kanthou et al., 100 µs PEFs (0.05–0.2 kV/cm, 3 p) caused minimal vimentin disruption to a confluent monolayer of HUVECs, despite significant actin and MT disruption [60]. However, some vimentin disruption was seen at the periphery of cells, and recovered within 2 h. Finally, Thompson et al. showed cortical lamin localized within the nucleus during PEF treatment (600 ns, 27.7 kV/cm) in CHO-K1 cells [76]. Disruption of the lamin cortex correlated with nuclear permeabilization. These studies show IF disruption can occur, but more research is needed to fully evaluate PEF-induced effects.

Septins are considered the fourth component of the cytoskeleton. These GTP-binding proteins can form into filaments and rings [111]. To date, no study has investigated septin disruption after PEFs. However, low-magnitude (<2.5 V/cm) fields applied at 100–300 kHz, known as tumor treating fields (TTFields), have been shown to interfere with septin localization during mitosis [112].

## 5. Mechanisms of Cytoskeletal Disruption

A complete understanding of the mechanisms of PEF-induced cytoskeletal disruption is still lacking. However, numerous mechanisms of disruption have substantial experimental and computational support. Direct mechanisms of disruption via interactions between the electric field and cytoskeletal proteins (or associated proteins) may include conformation changes, electrophoresis, and electromechanical effects. Secondary, downstream mechanisms may also lead to cytoskeletal disruption through cell swelling, elevated cytosolic calcium levels, ATP depletion, cell signaling, or other pathways. In the following section, we discuss support for various mechanisms of cytoskeletal disruption.

### 5.1. Actin—Direct Mechanisms

**Figure 4:**
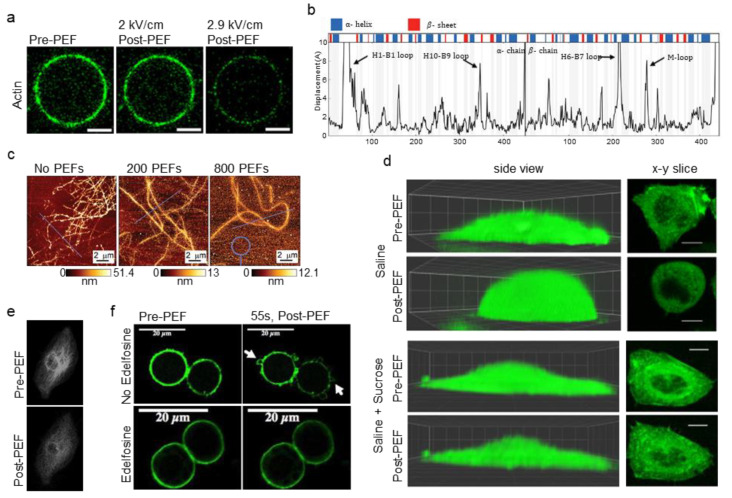
Mechanisms of cytoskeletal disruption. (**a**) Actin-encapsulated GUVs show decreased cortical actin fluorescence after PEF treatment, suggesting direct disruption of actin by electric fields. Scale bars 5 µm. Adapted from [72]. (**b**) Molecular dynamics simulations of tubulin show conformational changes to loops and side chains after nsPEF. Adapted from [84]. (**c**) Atomic force microscopy (AFM) imaging of polymerized, purified MTs after PEF treatment showed a loss of MT cylindrical structure as demonstrated by MT height. Adapted with permission from [81]. (**d**) Inhibiting swelling of Chinese hamster ovary (CHO-K1) cells maintained actin features after 600 ns PEFs (bottom). However, swelling caused a loss of actin structures and led to more homogenous actin structure (top). Scale bars 10 µm. Adapted with permission from [65]. (**e**) Loss of MTs in CHO-K1 cells occurred after 600 ns PEFs when the pulsation buffer contained calcium, even when swelling was mitigated. Adapted from [75]. (**f**) Treatment of CHO-K1 cells with edelfosine to inhibit PLC activity and PIP_2_ hydrolysis prevented blebbing after PEFs. Adapted with permission from [77].

Experiments with actin-encapsulated GUVs by Parrier et al. suggest that the actin cortex can be affected by electric fields through direct mechanisms such as electrophoresis and electromechanical stress [72]. Delivering consecutive pulses (500 µs, 0.1–10 kV/cm, 1–30 p) of increasing field strength to actin-encapsulated GUVs resulted in reduced fluorescence of the actin cortex, but not the diffuse fluorescence within the GUVs (Figure 4a). The decreased fluorescence of the cortex, which suggests a breakdown of actin, occurred over tens of seconds after the pulse and was slightly greater at the poles. Since biological processes can be excluded in GUVs, direct effects on actin are suggested. The study compared the approximate mechanical and electrophoretic forces experienced by an average actin filament and found that electrophoretic forces were likely four times larger than mechanical forces. Mechanical forces on the membrane are derived from Maxwell-stress induced bending and stretching of the lipid membrane. Due to these forces, empty GUVs take on prolate, oblate, or spherocylindrical shapes during PEF treatment and cause membrane bending and stretching [96,113]. This shape change, although significantly attenuated in cells and actin-encapsulated GUVs, may contribute to cortical actin disruption. In this study, however, the calculated force of mechanical disruption was below reported values for filament rupture or depolymerization, suggesting that electrophoretic forces had a major role in the actin cortex. Continued research with biomimetic GUVs of increasing complexity will further elucidate how these structures are involved in PEF treatments [114]. Alteration of actin-associated proteins by PEFs may also result in direct changes to actin dynamics, however, this has yet to be investigated.

### 5.2. Microtubules—Direct Mechanisms

Tubulin is a highly polar molecule and is highly negatively charged, especially at the c-terminus tail. Compared to all proteins of known structure, tubulin has a 4–5 times higher electrical charge (−22e average per monomer) and dipole moment (2166 Debye) than average [79]. These unique electrical properties make tubulin a target for direct modulation by external electric and magnetic fields. Numerous studies have experimentally demonstrated that purified MTs can migrate and align under electric fields [115,116,117,118,119,120,121]. Electric fields have also been shown to disrupt MT polymerization, which is exploited as the mechanism of action of tumor treating fields (TTFields) that use low-magnitude (<2.5 V/cm) fields applied at 100−300 kHz [122]. Molecular dynamics simulations also indicate that electric fields of GHz frequencies can disrupt tubulin and tubulin associated proteins [123,124,125]. Adding to the existing literature on MT dynamics under electric fields, recent molecular dynamics studies and experimental studies now demonstrate that PEFs can directly disrupt MTs.

Molecular dynamics simulations by Timmons et al. indicate conformational changes to tubulin after nsPEFs [84]. Simulation of a single 10 ns PEF (750 kV/cm) indicated conformational changes to charged and flexible regions of sidechains and loops of tubulin such as α: H1-B2 loop, β: M-loop, and c-termini (Figure 4b). Fields as low as 50 kV/cm caused the rearrangement of the α: H1-B2 loop in simulations. Since loop–loop interactions govern binding of adjacent heterodimers in MTs, changes to these regions could promote MT catastrophe (depolymerization). Conformational changes to tubulin may also affect a MTs ability to resist buckling. Additionally, intradimer curvature increased in simulations of PEF treatment. Intradimer curvature increases during depolymerization as protofilaments "peel" away from the MTs, so increased intradimer curvature after PEFs suggests PEFs-reduced MT stability. In another molecular dynamics study of tubulin by Marracino et al., simulation of a 30 ns PEF showed that fields of 200 kV/cm increased the dipole moment by 50%, and fields of 1 MV/cm increased the moment by three times [79]. Increased dipole moment may make the MT lattice unstable. This study did not show unfolding to tubulin’s secondary structure motifs up to 1 MV/cm, however electrostatic forces did pull the c-terminus tail away from the tubulin body. As the c-terminus is required for tubulin-associated protein interactions such as with motor proteins, this deformation may change MT dynamics after PEFs.

Experimental studies also indicate that PEFs can directly modulate MT dynamics. In a study by Chafai et al. with purified tubulin, MTs showed altered polymerization dynamics after PEFs (11 ns, 20 kV/cm, 100–800 p) [81]. Tubulin treated with 400 and 800 pulses did not polymerize to the same levels as untreated controls. At high fields, autofluorescence decreased following depolymerization of the MT, reflecting conformational changes. The zeta potential of tubulin (highly influenced by the c-terminus tail) decreased for 400 and 800 pulses before polymerization, but returned to control values after depolymerization. Immunoblotting did not reveal any damage to α-tubulin. MT structure, as measured by AFM, indicated changes after PEFs (Figure 4c). After 200 pulses, MTs showed decreased height indicating collapsed or open structures, while 800 pulses resulted in MTs of even lower height, suggesting open structures.

Modulation of MT-associated proteins/structures such as MT motor proteins, MT severing enzymes, MT-associated MAP proteins, and altered MT-membrane interactions may also alter MT dynamics. For example, molecular dynamics simulations by Průša et al. of the motor protein kinesin-I docked on a single tubulin heterodimer indicated that a 30 ns PEF (1 MV/cm) can alter the kinesin dipole moment (magnitude and angle), affect the contact surface area between kinesin and tubulin, and alter important structures such as MT binding motifs and nucleotide hydrolysis sites [80]. These nsPEF-induced changes to kinesin may result in altered MT dynamics. Furthermore, nsPEFs have been shown to generate acoustic shock waves [126] that may disrupt MT in a similar manner as ultrasound [47,127,128]. However, more research is needed to evaluate these pathways of disruption.

### 5.3. Swellling/Volume Change

Cell swelling is a common response to PEFs, typically manifested by cell blebbing and rounding [53,54,59,65,67,69,75,77]. PEFs permeabilize the cell membrane to small solutes but not to larger solutes, creating colloid-osmotic pressure that drives water into the cell. To block cell swelling, sucrose or other large molecules such as polyethylene glycol can be added to the pulsation buffer as these large molecules cannot enter the permeabilized cell and can balance colloid-osmotic forces. The cytoskeleton is structurally and functionally linked with various membrane transporters, which together actively regulate cell volume [129]. Given the interdependent relationship between cell volume and the cytoskeleton, swelling after PEFs can cause cytoskeletal changes.

An example of swelling-induced cytoskeletal breakdown was reported by Pakhomov et al. after 600 ns PEFs (1.92 kV/cm, 4 p) were applied to CHO-K1 cells (Figure 4d) [65]. In a buffer that did not inhibit PEF-induced swelling, nsPEFs caused cell rounding and led to partial disassembly of actin fibers and non-filamentous actin "patches". However, buffer with added sucrose to block colloid-osmotic swelling prevented the disassembly of actin structures, implicating swelling as the cause of actin disruption. As another example, Rassokhin et al. showed that during PEF treatment (60 ns, 10 kV/cm, >1000 p) of human monocytes (U-937 cells), inhibiting colloid–osmotic swelling via sucrose inhibited pseudopod-like bleb formation, implicating water influx in their formation [67].

Despite strong evidence that swelling drives cytoskeletal disruption, several other studies indicate cytoskeletal disruption in the absence of cell swelling [47,55], suggesting additional mechanisms are involved.

### 5.4. Cytosolic Calcium Concentration

The concentration of free calcium in the cytosol is around 10^−4^ mM, more than 10,000 times less than the extracellular concentration under normal conditions (~1–2 mM) [130]. Low cytosolic calcium is maintained by calcium pumps that use ATP to pump calcium outside the cell or inside the endoplasmic reticulum (ER). When PEFs disrupts the cells membrane, extracellular calcium can enter the cell by diffusion through membrane pores or passage across voltage-gated calcium channels. Even in the absence of extracellular calcium, cytosolic calcium levels can increase after PEFs due to permeabilization of the ER, which contains calcium concentrations typically at 0.1–0.8 mM [131]. Calcium is a potent signaling molecule, and modulates the cytoskeleton including both actin and MTs. Table 1 indicates whether studies on PEF-induced cytoskeletal disruption included calcium in the pulsation buffer.

High cytosolic levels of calcium have been shown to depolymerize MTs and to affect actin filaments [132,133,134]. For example, Downey et al. showed that electropermeabilized neutrophils had a breakdown of f-actin in buffers with calcium concentrations greater than normal intracellular levels (~100 nM) [71]. Similarly, Harkin et al. found that pulsation buffer containing calcium chloride levels above 100 µM caused MT disruption in chick embryo corneal fibroblasts after PEFs (10–20 ms, 0.5–1.0 kV/cm, 1 p) [68]. Serum-free media (calcium concentration ~1mM) used as the pulsation buffer caused MT disruption and inhibited migration in cells. Contrarily, cells displayed normal migration and MT structure after PEFs in buffer containing low amounts of calcium (1 µM). In another study, Thompson et al. found that cells treated with 600 ns PEFs (16.2 kV/cm, 20 p) showed MTs depolymerization when the pulsation buffer contained calcium, even when cell swelling was mitigated (Figure 4e) [75]. MT disruption was less pervasive in calcium-free media. As a final example, Titushkin et al. found that even low-level electric fields (2 V/cm) caused cytoskeletal disruption such as decreased YM and activated-ERM proteins that were likely caused by calcium influx [135].

Calcium independent MT disruption, however, is also reported. For example, Carr et al. showed that nsPEFs (10 ns, 44 kV/cm, 100 p) applied to human glioblastoma (U-87 GM) cells caused a breakdown of MTs without increased cytosolic calcium levels, as monitored by Fluo-4 AM fluorescence [47]. Thus, calcium appears to be one of many pathways for cytoskeletal disruption. Calcium modulates many pathways, and thus calcium influx may lead to a diversity of cell responses that together disrupt the cell cytoskeleton.

### 5.5. ATP Depletion

Restoration of ion concentrations (such as calcium) after PEF disruption is an energetically expensive process that is accomplished by ATP-powered pumps such as Na⁺/K⁺-ATPase and Ca^2+^ ATPase. ATP consumption by ion pumps combined with metabolite leakage through membrane pores can result in severe ATP depletion [51,136,137]. ATP depletion has been shown to alter cytoskeletal dynamics [138,139] (although it may have little effect on cell mechanical properties [140]), and thus PEF-induced ATP depletion may be a factor in cytoskeletal response. In one study by Titushkin et al., non-electroporating direct current (2 V/cm, 60 min) applied to osteoblasts increased the tether length of the cell membrane similar to cells with chemically-depleted ATP [135]. ATP depletion inhibited linker proteins and caused membrane separation from the cytoskeleton. However, as mentioned previously, Rols et al. showed that pre-incubation of cells with ATP and GTP did not affect cell membrane resealing [62]. Additional studies are needed to determine the contributions of ATP depletion to cytoskeletal disruption.

### 5.6. Additional Mechanisms

A recent paper by Tolstykh et al. implicates the depletion and hydrolysis of the lipid signaling molecule phosphatidylinositol-4-5-bisphosphate (PIP_2_) as a root cause of cell swelling and blebbing after nsPEFs [77]. PIP_2_ is important for a number of signaling pathways, and regulates ion channels, modulates cell volume, and binds to many actin regulatory proteins that control actin dynamics. After 600 ns PEFs (16.2 kV/cm, 20 p), PIP_2_ depletion was observable 2 seconds after pulsing. The poles of the cells showed the most PIP_2_ depletion, the same regions that showed the most pronounced blebbing. Dimming of the actin cortex occurred and blebbing began by 9 seconds, the same time that peak phospholipase C (PLC) activity was detected in a previous study [141]. Pretreatment of cells with edelfosine to block PLC activity and prevent PIP_2_ hydrolysis significantly reduced cell perimeter changes and eliminated blebbing for treatment of a single pulse (Figure 4f). These results suggest PIP_2_ depletion and PLC activation initiate a pathway that causes membrane dissociation from the actin cortex and leads to cell swelling and blebbing.

In addition to the numerous mechanisms outlined above, other mechanisms may also contribute to cytoskeletal disruption including intracellular protein release [142], pH changes [66], generation of reactive oxygen species, activation of membrane-bound receptors, stretch-activated cation channels, or possibly many others. Caspase activity after PEFs causes cytoskeletal breakdown, however caspase activity indicates apoptosis [51]. Significant opportunities still exist for identifying the complete pathways of cytoskeletal disruption.

### 5.7. Disruption Mechanisms and Pulse Length

While most observed features of cytoskeletal disruption seem to be conserved across pulse lengths ranging from nsPEFs to msPEFs, it seems likely that unique disruption characteristics may arise from particular pulsing parameters (length and magnitude). Given the available studies, however, it is difficult to make a proper assessment of the differences in cytoskeletal disruption between PEFs of various pulse lengths (nsPEFs, µsPEFs, and msPEFs). A few studies investigate more than one pulse length [70,76], however, essentially no studies have provided a comprehensive analysis of cytoskeletal response across a wide range of pulse lengths. Several studies do investigate cytoskeletal disruption using the same cell type (CHO-K1, for example), however, direct comparison is complicated by varying experimental conditions and analysis methods between studies. Future studies that maintain constant experimental conditions (e.g., same cell type, same pulsing media, same analysis method) and test a range of pulse lengths may more clearly identify unique characteristics of disruption based on pulse length.

Despite the current lack of studies comparing differences in cytoskeletal responses across pulse lengths, our current understanding of the mechanisms of disruption suggests that some mechanisms may be unique to (or more prominent at) certain pulse lengths and magnitudes. For example, direct modulation of cytoskeletal (or cytoskeletal-associated) proteins by conformational changes likely are limited to PEFs of very high field strengths such as those used during nsPEFs (>10 kV/cm). Longer µsPEFs and msPEFs, however, may more readily enable cytoskeletal breakdown by electrophoresis and electrodeformation due to the longer duration of the applied field. (Millisecond PEFs are widely used for gene transfection due to their electrophoretic effects [143]). Longer pulses also generate larger pores than nsPEFs [9,38,39], which may enable the leakage of larger cytoplasmic molecules. Leakage of cytoplasmic molecules (proteins, ATP, GTP, etc.) may lead to cytoskeletal disruption. Other mechanisms of disruption, however, such as calcium influx and colloid–osmotic swelling, likely occur across pulse lengths ranging from nsPEFs to msPEFs. PEFs ranging from nanoseconds to milliseconds can disrupt the lipid membrane and ion channels that together alter intracellular ion concentrations to cause cytoskeletal disruption. Likewise, membrane and ion disruption can lead to volume changes that alter the cytoskeleton for various pulse lengths. Beyond these insights on how pulse length may affect cytoskeletal disruption, additional studies are needed to more precisely determine the predominant mechanism(s) for various pulse lengths.

## 6. Cell-Matrix and Cell–Cell Junction Disruption

A critical function of cytoskeletal filaments is to interact mechanically and biochemically with a cell’s environment via transmembrane adhesion complexes [144]. Adhesion complexes, such as focal adhesions, link the cytoskeleton to the cell’s environment, which in vivo is the extracellular matrix. Cell-to-substrate interactions are responsible for cell shape, migration, signaling, differentiation, and cell function [36,145]. Likewise, cell-to-cell junctions such as adherens junctions and tight junctions establish mechanical stability, enable cell–cell signaling, and limit molecular transport between adjacent cells (paracellular transport) [146,147]. Both cell-to-cell and cell-to-substrate interactions are disrupted by PEFs.

### 6.1. Cell-Matrix Disruption

Cell-substrate disruption is commonly reported after PEF treatment in the form of cell rounding and shape change [49,55,56,59,65,66]. Cell rounding may occur to accommodate changes due to cell swelling, or be due to breakdown of adhesion sites. Loss of cell adhesion is temporary after PEF treatment (except for cell death), and PEFs do not induce tumorigenic characteristics in cells such as anchorage-independent growth [55]. Adherent cells typically remain adherent after PEF treatment despite some rounding, however in one study complete detachment of cells was reported [49].

Adhesion after PEFs is likely cell-type dependent. Pehlivanova et al. used a crystal violet assay to show that the adhesive behavior of two human breast cancer cell lines (MDA-MB-231, MCF-7) and a mouse fibroblast cell line (NIH/3T3) is cell-type and field-strength dependent after biphasic PEFs (50 µs + 50 µs, 0.2–1 kV/cm, 8 p) [56]. A similar finding of cell-type dependent adhesion was indicated in another study by Szewczyk et al. Zyxin, a protein involved with focal adhesions, showed increased expression in healthy mouse myoblasts (C2C12 cells) after 100 µs PEFs (1 kV/cm, 8 p), but decreased expression in human rhabdomyosarcoma (RD) cells, indicating altered cell–substrate connections [73]. Harkin et al., found that chick embryo corneal fibroblast migration, which requires cell–substrate interactions, did not occur in the first 2 h after millisecond PEFs (10–20ms, 0.5–1.0 kV/cm, 1 p) in serum-free media [68].

Some studies have suggested that adherent cells with highly developed cytoskeletons may be more resistant to PEFs than cells in suspension [49,148], however more studies are needed to support or refute this hypothesis.

### 6.2. Cell-Cell Junction Disruption

Cell-to-cell disruption is commonly studied in vitro with cell monolayers, as cell–cell junctions limit monolayer permeability by connecting adjacent cells. Adherens junctions and tight junctions link the actomyosin contractile systems of adjacent cells via linker proteins to transmembrane proteins, such as vascular endothelial cadherin (VE-cadherin) [146,147]. Several studies show that the permeability of endothelial monolayers increases after electroporation. Kanthou et al. showed that VE-cadherin fluorescence decreased in a human umbilical vein endothelial cell (HUVEC) monolayer after PEFs (100 µs, 0.05–0.2 kV/cm, 3 p) [60]. Cytoskeletal and junctional disruption resulted in increased permeability during the first 30 min after PEFs. Likewise, Meulenberg et al. showed increased permeability of a human dermal microvascular endothelial cell (HMEC-1) monolayer after PEFs and ECT [53]. ECT induced more rapid cell-to-cell disruption than PEFs alone, and decreased cell turgidity while increasing cell–cell gaps. Numerous other in vitro studies demonstrate increased monolayer permeability and cell–cell junction disruption after PEFs [57,149,150,151,152,153,154]. Electrofusion of adjacent cells [53,155] can also occur during PEF treatment, and the cytoskeleton likely plays a key role in this process [156,157].

## 7. Considerations for Electroporation Therapies

Cytoskeletal disruption is principally manifested as anti-vascular effects after in vivo PEF therapies. Healthy microvascular is maintained by a balance of intracellular forces generated by the cytoskeleton of endothelial cells, and extracellular forces transferred cell–cell and cell–matrix across adhesion complexes [158]. Alteration of actin and MT dynamics, therefore, can cause significant endothelial barrier dysregulation. Studies show that inhibition of actin or MT polymerization results in increased barrier permeability [159,160,161,162] and capillary collapse [163]. Furthermore, increases in endothelial contractility via actomyosin stress fibers results in enlarged cell–cell gaps and leaky vessels [164,165].

PEFs ranging from nanoseconds to milliseconds cause significant decrease of blood flow to treated tissues, a phenomenon known as the vascular lock effect [166,167]. This effect has been well studied in 100 µs ECT therapies. After 100 µs PEFs, blood flow reduces to near zero flow within seconds. This rapid, but transient reduction of blood flow is attributed to vasoconstriction of afferent arterioles, mediated by the sympathetic nervous system in response to electroporation of muscle and/or vascular endothelial cells [167]. Within minutes, a second mode of disruption becomes prominent: cytoskeletal and cell–cell junction disruption. As has been already described, significant alteration of the cell cytoskeleton occurs within minutes after PEFs treatment. Endothelial cells may be particularly susceptible to PEFs and ECT [168,169], thus enhancing barrier dysregulation. Loss of barrier integrity leads to extravasation of fluids, increased interstitial fluid pressure, and decreased intravascular pressure that together result in decreased perfusion. In addition, endothelial cell swelling physically obstructs vessels leading to increased vascular resistance and decreased perfusion [169]. Without the addition of chemotherapy, effects of reversible electroporation can last up to 24 h. ECT treatment, however, causes a permanent loss of blood flow due to endothelial cell death.

nsPEFs also cause profound and prolonged blood flow disruption [8,170,171,172,173]. A study by Bardet et al. showed that nsPEFs as short as 10 ns transiently decreased capillary diameter, caused capillary collapse, and decreased perfusion [171]. Other studies show nsPEFs destroy the capillaries feeding tumors within about one day, and cause extended loss of perfusion for greater than two weeks [8,170].

The anti-vascular effects of ECT are clinically useful, as the vascular lock prevents convection of adjuvant drugs (calcium, chemotherapy) away from the treatment area. ECT is also effective for eliminating bleeding from of bleeding tumors [167] and needle insertion sites. Reduced blood flow also induces localized hypoxia in the treatment area, which can be key for promoting tumor cell death.

PEF-induced vascular hyperpermeability may also be exploited for the treatment of brain tumors. The blood-brain barrier (BBB) creates a neuroprotective environment for the brain by strictly regulating the transport of substances into the brain through a variety of specialized transporters and cell–cell tight junctions. Many chemotherapies show limited ability to cross this barrier in clinically-effective doses [174,175]. PEF-induced BBB disruption should, therefore, provide a therapeutic benefit when applied in conjunction with chemotherapies. In vivo studies show that PEF application to the brain can disrupt the BBB [176,177,178,179,180]. BBB disruption may even occur at sub-electroporation thresholds [154], suggesting that PEF treatment of brain tumors may enable irreversible electroporation of the tumor bulk within a large region of reversible BBB disruption. Clinical studies will be required to assess the use of PEFs therapies for the treatment of brain cancers, but preclinical studies show significant promise.

### Cytoskeletal Targets for Improved PEF Therapies

Malignant cells show distinct cytoskeletal alterations from healthy cells, and an improved understanding of how the cytoskeleton is involved with PEF therapies may lead to new opportunities to exploit differences between healthy and cancerous cells. Many PEF studies show differential responses between healthy cells and cancerous cells [48,181,182,183], and cytoskeletal differences may be a contributing factor of this response. For example, Pehlivanova el al. showed that under the same PEF treatment, human breast cancer cells and mouse fibroblasts had different cytoskeletal responses—structure was conserved in fibroblasts while cancer cells showed a loss of their cytoskeleton [56]. Malignant cells adapt their cytoskeleton for proliferation and infiltration and generally have reduced stiffness [184,185]. Given the differences between healthy and cancerous cells, futures studies may show ways to preferentially target malignant cells based on their cytoskeleton.

Many anti-cancer drugs, such as paclitaxel, target MTs since these structures are critical for cell division. PEFs may find synergy with taxane-based cancer therapies to modulate MT dynamics to alter drug binding to tubulin [79]. Some cancer cells show altered γ-tubulin levels, which modulate MT nucleation. Thus, modulating MT nucleation by PEFs may open new therapeutic opportunities [82]. Electromechanical models of mitotic spindle vibration by Havelka et al. suggest nsPEF-driven electro-acoustic behavior of mitotic spindles may have a disrupting effect on kinetochore-microtubule binding or chromatid separation with implications for cancer treatment [186]. Actin disruption can cause mitochondrial disfunction, and thus PEF-induced actin disruption may prove useful for inhibiting mitochondrial function for cancer therapies [187]. Further research may reveal additional cytoskeletal targets for cancer therapy.

## 8. Conclusions

Considering the cellular response to PEFs from a cytoskeletal perspective reveals a complex, multi-factor process of cytoskeletal disruption. Given the available data, cytoskeletal response to PEFs is undoubtedly influenced by cell type, pulse parameters (pulse length, pulse magnitude, pulse number), and pulsation media. Direct effects on the cytoskeleton, such as protein conformation changes, electrophoretic effects, and electromechanical effects are accompanied by downstream effects to produce observed cell morphology and behavior. Downstream mechanisms include swelling, calcium influx, ATP depletion, PIP_2_ depletion, and likely others. Additional research is required to determine which mechanisms are predominant. Cytoskeletal disruption is chiefly manifested in PEF therapies by anti-vascular effects of treated tissues. Further research is needed to evaluate the possibility of new targets based on cytoskeletal dynamics for improved PEF therapies. Insight into the exact cause of cytoskeletal disruption and the mechanisms of cytoskeletal recovery may prove useful for improving tumor cell death and PEF treatment selectivity.

## Figures and Tables

**Table 1:** Summary of studies on cytoskeletal disruption by pulsed electric fields.

Study (Year)	Cell Type (A: adherent; S: suspension; M: monolayer)	Pulse Length	Field Strength (kV/cm)	Pulse # (freq)	Pulsation Buffer (with(+) or without(−) Ca^+2^)	Cytoskeletal Agents	Focus	Outcomes
Harkin et al. (1996) [68]	Chick embryo corneal fibroblasts (A)	10–20 ms ^1^	0.5, 0.625, 0.75, 0.875, 1.0	1 (N/A)	Basal Media (+ Ca^+2^); Buffers (+/− Ca^+2^)		Actin MT IF	Media as pulsation buffer inhibited migration for 2 h, caused MT loss after 10 min, but showed MT recovery in 3–4 h; Some buffers preserved migration and MTs, excepted with high concentrations of CaCl. Extracellular calcium adversely affects cell migration due to MT disruption. Staining showed no impact to actin. Perinuclear collapse of IFs, with recovery in 3–4 h.
Chopinet et al. (2013) [59]	CHO wild type (A)	5 ms	0.4	8 (1 Hz)	Buffer (− Ca^+2^)		Actin	AFM measurements showed YM decreased 40% after PEFs; YM more spatially homogeneous within 1 min; YM similar for electrode-facing regions and perpendicular-facing regions; Membrane rippling, loss of actin fibers 3–15 min; YM not correlated with cell resealing time; Cell swelling present; Cells re-spread by 23 min.
Chopinet et al. (2014) [69]	CHO wild type (A)	5 ms	0.4	8 (1 Hz)	Buffer (− Ca^+2^)	LatB LatA	Actin	AFM showed YM of CHO cells decreased 30% by LatB, and recovered in 35 min after drug removal; Magnitude and duration of YM response are similar between PEF treatment and recovery from LatB; Cells do not recover from LatA and PEFs. PEFs before LatB treatment showed additive effects.
Hohenberger et al. (2011) [70]	BY-2 ^2^ (S)	3 ms; 10 ns	0.8, 1.6; 33	1–10, (1 Hz); 1–20, (NR)	Buffers (+/− Ca^+2^)		Actin MT	Genetically modified BY-2 cells with increased actin bundling showed less PI uptake; Actin bundling stabilized the cell membrane against permeabilization after msPEFs and nsPEFs.
Downey et al. (1990) [71]	Human Neutrophils (S)	NR ^3^	NR	2 (NR)	Buffer (+ CA^+2^)		Actin	Influx of extracellular calcium post-PEFs caused depolymerization of f-actin.
Perrier et al. (2019) [72]	Actin-GUV; Empty-GUV	500 µs	0.1–3, 3–10	1–30, 2–4 (0.017 Hz)	Buffer (− Ca^+2^)		Actin	Actin-GUVs had increased and prolonged dye uptake compared to empty-GUVs; Actin-GUVs had reduced electrodeformation; Actin cortex fluorescence decreased after PEFs; Electrophoretic effects on actin calculated to be 4× greater than electrodeformation effects.
Rols et al. (1991) [63]	CHO-WTT (A)	100 µs	1.5	10 (1 Hz)	Buffer (− Ca^+2^)	CytB, COL, ATP, GTP	Actin MT	Pretreatment with COL decreased resealing time and electrofusion rate post-PEFs; CytB had no significant change on electrofusion rates.
Rols et al. (1992) [61]	CHO-WTT (A) RBC (S)	100 µs	1.8, 2.4	10 (1 Hz)	Buffer (− Ca^+2^)	CytB, COL, ATP, GTP	Actin MT	COL-treated cells resealed 3× faster; ATP/GTP in buffer did not affect resealing time; Pore resealing, but not pore formation, affected by cytoskeleton; Microvilli density increased post-PEFs.
Teissie et al. (1994) [62]	CHO-WTT (A) RBC (S)	100 µs	1.8, 2.4	10 (1 Hz)	Buffer (− Ca^+2^)	CytB, COL, ATP, GTP	Actin MT	COL-treated cells resealed 3x faster; Microvilli density increased post-PEFs; Extracellular ATP increased microvilli length; Resealing rate was dependent on MTs.
Kanthou et al. (2006) [60]	HUVEC (M)	100 µs	0.05, 0.1, 0.15, 0.2	3 (1 Hz)	Basal Media (+ Ca^+2^)		Actin MT IF	Actin and MTs depolymerized in 5 min; Actin became honeycomb-like; MTs fragmented; Burst of pMLC at 30 and 60 min; Cytoskeletal recovery 1–2 h; IFs relatively unchanged, except at cell periphery.
Meulenberg et al. (2012) [53]	HMEC-1 (M)	100 µs	0.068, 0.137, 0.274, 0.411, 0.548, 0.685	8 (1 Hz)	Buffer (− Ca^+2^)		Actin MT	Actin stress fibers thinned, fragmented, and took on a honeycomb-like organization; ECT caused cell shrinkage; MTs became densely packed, less extended, and fragmented; Partial monolayer recovery at 24 h for PEFs, but no recovery for ECT; Cell swelling by 10 min; Cell edges ruffled at 2 h; ECT caused more rapid increase in membrane permeability.
Szewczyk et al. (2018) [73]	C2C12 (A, S) RD (A, S)	100 µs	0.6, 0.8, 1	8 (1 Hz)	Buffers (+/− Ca^+2^)		Actin	Ca^+2^ in buffer increased zyxin expression and actin stress fiber tension in normal C2C12 cells, but decreased zyxin expression and depolymerized actin in cancerous RD cells; Zyxin changes indicated altered cell–cell and cell–substrate connections; Adherent cells showed higher viability after PEFs than suspended cells.
Kim et al. (2020) [74]	NCI-H640 (A) MCR-5 (A)	100 µs	0.3, 0.5, 0.7, 1	8 (10 Hz)	Basal Media (+ Ca^+2^)	CytD	Actin	CytD pretreatment decreased PI uptake after PEFs compared to PEFs alone; Annexin V-FITC signal decreased with low concentrations of CytD and low field strengths.
Pehlivanova et al. (2012) [56]	MDA-MB-231 (A); MCF-7 (A); NIH/3T3 (A)	Bipolar: 50–20–50 µs	0.2, 0.5, 1.0	8 (1 Hz)	Basal Media (+ Ca^+2^)		Actin	Adhesion post-PEFs was cell-type and field-strength dependent; More cytoskeletal disruption in cancerous cells than fibroblasts; Stress fibers were thinner, fewer, and at high fields located peripherally; Podosomes formed; Actin recovered in 24–48 h, except at high fields.
Pakhomov et al. (2014) [65]	CHO-K1 (A)	600 ns	1.92	1, 4 (2 Hz)	Buffer (− Ca^+2^)		Actin	Mitigating cell swelling prevented actin disruption post-PEFs. Without mitigating swelling, cells showed increased fluorescence of diffuse actin, reduced bright spots, and reduced overall actin fluorescence.
Thompson et al. (2014) [75]	CHO-K1 (A)	600 ns	16.2	1, 20 (NR)	Buffers (+/− Ca^+2^)	PTX	MT	Ca^+2^ in buffer caused MT disruption and halted lysosome transport; MT disruption occured despite mitigating blebbing and swelling; PTX stabilized MTs against depolymerization after PEFs.
Thompson et al. (2016) [76]	CHO-K1 (A)	600 ns; 10 ns	27.7; 150	1, 5, 10, 20 (1 Hz)	Buffers (+/− Ca^+2^)		IF	Localization of cortical lamin within the nucleus after PEFs; Disruption of lamin cortex correlated with nuclear permeabilization.
Tolstykh et al. (2017) [77]	CHO-K1 (A)	600 ns	16.2	1, 20; (5 Hz)	Buffer (+ CA^+2^)		Actin	PIP_2_ depletion and PLC activity led to cell swelling and blebbing; Edelfosine to block PLC activity inhibited blebbing.
Xiao et al. (2011) [78]	HepG2 (A)	450 ns	8	30 (1 Hz)	NR	CytB	Actin	CytB treatment before PEFs decreased necrotic and apoptotic cells; CytB alone did not decrease viability compared to controls.
Ford et al. (2010) [51]	B16-F10 (S)	300 ns	12, 18, 26, 40, 60	1, 3, 10 (NR)	Buffer (− Ca^+2^)		Actin	Caspase activity and cytoskeletal integrity mutually exclusive; ATP decreased after nsPEFs.
Steuer et al. (2016) [57]	WB-F344 (M)	100 ns	15,20	20 (NR)	Complete Media (+ Ca^+2^)		Actin	F-actin fragmented, less organized, and depolymerized after PEFs; cell morphology generally unchanged; Partial actin recovery by 60 min.
Steuer et al. (2017) [55]	WB-F344 (M); WB-Ras (M) ^4^	100 ns	20	20 (NR)	Complete Media (+ Ca^+2^)		Actin	AFM showed >30% decrease in YM after 8 min; Actin fibers shorter, less aligned at 5 min; increased diffuse fluorescence at 15 min; YM recovered to control values at 13–28 min; Partial recovery 30–60 min; PEFs did not induce tumorigenic behavior.
Stacey et al. (2011) [49]	Jurkat (S) HeLa (A) SV40 (A)	60 ns	60	1 (N/A)	Buffer (NR)	CytB	Actin	Adherent cells had ruffled membranes and rounded up with speckled actin spots; Jurkat cells showed actin speckling; Decreased viability in HeLa and SV40 cells after pretreatment with CytB.
Rassokhin et al. (2011) [67]	U-937 (A)	60 ns	10	>1000 (10–20 Hz)	Buffer (− Ca^+2^)	CytD	Actin	Pseudopod-like bleb (PLB) growth toward the anode during PEFs; CytD prevented PLBs; Actin caused unique shape; Inhibiting cell swelling prevented PLBs; Not replicated in CHO, Jurkat, or GH3 cells.
Dutta et al. (2015) [64]	Jurkat Clone E6-1 (S)	60 ns	15, 60	1 (N/A)	Complete Media (+ Ca^+2^)		Actin	AFM showed 53% decrease in YM after 15 kV/cm PEFs and minimal actin/morphological changes; At 60 kV/cm, YM decreased 85%; Cell shape changed, peripheral actin became more diffuse, and actin foci formed.
Marracino et al. (2019) [79]	N/A	30 ns	200, 500, 1000	1 (N/A)	N/A		MT	MD simulations showed tubulin dipole moment increased 50% at 200 kV/cm and 300% at 1 MV/cm; No unfolding of structural motifs, but C-terminus tail pulled away from tubulin body.
Průša et al. (2019) [80]	N/A	30 ns	1000	1 (N/A)	N/A		MT	MD simulations of kinesin-I docked to a tubulin heterodimer indicated altered kinesin dipole properties, altered contact surface area between kinesin and tubulin, and altered structures including MT binding motifs and nucleotide hydrolysis sites.
Chafai et al. (2019) [81]	N/A	11 ns	20	100, 200, 400, 800 (1 Hz)	Buffer (− Ca^+2^)		MT	Purified tubulin showed decreased polymerization after PEFs; Autofluorescence measurements suggested conformational changes after PEFs; Altered zeta potential of tubulin after PEFs; AFM showed altered tubulin structures after PEFs; Immunoblots showed no damage to tubulin.
Havelka et al. (2019) [82]	RBL-2H3 (A)	11 ns	~67.5	4000 (100 Hz)	Buffer (+ Ca^+2^)		MT	GFP-tagged MT end-tracking protein EB3 showed decreased fluorescence and size after PEFs.
Thomson et al. (2013) [83]	CHO-K1 (A) U-937 (S) Jurkat Clone E6-1 (S)	10 ns	150	100 (NR)	Complete Media (+ Ca^+2^)	PTX, JAS, LatA, NOC	Actin MT	LatA pretreatment decreased CHO-K1 elasticity to levels of Jurkat cells, however CHO-K1 cells had higher viability after PEFs; MT disruption by NOC decreased PI uptake and Annexin V-FITC fluorescence; LatA pretreatment increased PI uptake and Annexin V-FITC; JAS and PTX pretreatment did not change membrane damage after PEFs.
Berghöfer et al. (2009) [58]	BY-2 (S)	10 ns	33	1 (N/A)	Buffer (− Ca^+2^)	PHD	Actin MT	Depolymerization of cortical actin; Detachment of transvacuolar actin bundles from cell periphery; Actin contraction toward the nucleus; PHD pretreatment decreased uptake of trypan blue and suppressed actin detachment from cell periphery; MTs affected within 1 min, and maximally disordered by 3 min.
Thomson et al. (2014) [54]	CHO-K1 (A)	10 ns	150	50, 100 (1 Hz)	Complete Media (+ Ca^+2^)	LatA	Actin	AFM showed that YM of newly-adherent cells decreased ~50% after PEFs and caused partial loss of the actin cortex; LatA caused ~80% decrease in YM and fully disrupted the actin cortex; LatA treatment before PEFs increased PI uptake and decreased viability.
Carr et al. (2017) [47]	U-87 MG (A)	10 ns	44	100 (10 Hz)	Buffers (+/− Ca^+2^)		MT	MTs showed buckling, breaking, depolymerization; MT end-tracking protein EB3 showed altered dynamics post-PEFs. Decreased tubulin and EB3 comet fluorescence after PEFs; Decreased number of EB3 comets, but comet length increased; MT disruption independent of intra/extracellular calcium; MT disruption temporally linked with mitochondria depolarization.
Timmons et al. (2018) [84]	N/A	10 ns	50–750	1 (N/A)	N/A		MT	MD simulations indicated conformational changes to charged and flexible regions of sidechains and loops of tubulin such as α: H1-B2 loop, β: M-loop, and c-termini. Intradimer curvature increased in simulations after PEFs.

**Abbreviations:** AFM atomic force microscopy, ATP Adenosine Triphosphate, COL colchicine, CytB cytochalasin B, CytD cytochalasin D, ECT electrochemotherapy, freq frequency, GTP Guanosine Triphosphate, GUV giant unilamellar vesicle, IF intermediate filaments, JAS jasplakinolide, LatA latrunculin A, LatB latrunculin B, MD molecular dynamics, MT microtubules, NOC nocodazole, PEFs pulsed electric fields, PHD phalloidin, PI propidium iodide, PIP2 phosphatidylinositol 4,5-bisphosphate, PLC phospholipase C, pMLC phosphorylated myosin light chain, PLB pseudopod-like bleb, PTX paclitaxel, YM Young’s modulus. Cell Types: B16-F10 mouse melanoma, C2C12 mouse myoblasts, CHO Chinese hamster ovary cells (CHO wild type, CHO-K1, CHO-WTT clone), HeLa human cervical cancer (adenocarcinoma), HepG2 human hepatocellular carcinoma, HMEC-1 human dermal microvascular endothelial cells, Jurkat Clone E6-1 human T lymphocytes, MCF-7 human breast cancer (adenocarcinoma), MDA-MB-231 human breast cancer (adenocarcinoma), MRC-5 human lung fibroblasts, NCI-H460 human lung carcinoma, NIH/3T3 mouse fibroblasts, RBL-2H3 rat basophilic cells, RD human rhabdomyosarcoma, SV40 immortalized fibroblasts, U-87 MG human glioblastoma, U-937 human monocytes, WB-F344 rat liver epithelial cells. ^1^ Exponentially decaying pulse (125–960 uF); Time constant 10–20 ms. ^2^ In addition to wild type BY-2, BY-2 were genetically engineered to overexpress actin-binding domain 2 of plant fimbrin or with inducible expression of actin-bundling WLIM1 protein. ^3^ Exponentially decaying pulse (25 uF); Time constant not reported. ^4^ WB-Ras derived from WB-F344 with H-Ras oncogene.

**Table 2:** Agents used to disrupt the cytoskeleton.

**Actin**	**Inhibit Polymerization**	**Stabilize Polymerization**	**Studies Used**
Cytochalasin B or D (CytB/CytD)	X		[49,61,62,67,74,78]
Latrunculin A or B (LatA/LatB)	X		[54,69,83]
Phalloidin (PHD)	X		[58]
Jasplakinolide (JAS)		X	[83]
ATP		X	[61,62]
**Microtubules**	**Inhibit Polymerization**	**Stabilize Polymerization**	**Studies Used**
Colchicine (COL)	X		[61,62]
Nocodazole (NOC)	X		[83]
Paclitaxel (PTX)		X	[75,63]
GTP		X	[61,62]

## Abbreviations

AFM	atomic force microscopy
ATP	Adenosine Triphosphate
COL	colchicine
CytB	cytochalasin B
CytD	cytochalasin D
ECT	electrochemotherapy
ER	endoplasmic reticulum
freq	frequency
GET	gene electrotransfer
GTP	Guanosine Triphosphate
GUV	giant unilamellar vesicle
IF	intermediate filaments
IRE	irreversible electroporation
JAS	jasplakinolide
LatA	latrunculin A
LatB	latrunculin B
MD	molecular dynamics
MT	microtubules
NOC	nocodazole
PEFs	pulsed electric fields
PHD	phalloidin
PI	propidium iodide
PIP2	phosphatidylinositol 4,5-bisphosphate
PLC	phospholipase C
pMLC	phosphorylated myosin light chain
PTX	paclitaxel
p	pulses
YM	Young’s modulus
**Cell Types**	
B16-F10	mouse melanoma
C2C12	mouse myoblasts
CHO	Chinese hamster ovary cells (CHO wild type, CHO-K1, CHO-WTT clone)
HeLa	human cervical cancer (adenocarcinoma)
HepG2	human hepatocellular carcinoma
HMEC-1	human dermal microvascular endothelial cells
Jurkat	Clone E6-1 human T lymphocytes
MCF-7	human breast cancer (adenocarcinoma)
MDA-MB-231	human breast cancer (adenocarcinoma)
MRC-5	human lung fibroblasts
NCI-H460	human lung carcinoma
NIH/3T3	mouse fibroblasts
RBL-2H3	rat basophilic cells
RD	human rhabdomyosarcoma
SV40	immortalized fibroblasts
U-87 MG	human glioblastoma
U-937	human monocytes
WB-F344	rat liver epithelial cells.
